# Reprogramming resistance: phage-antibiotic synergy targets efflux systems in ESKAPEE pathogens

**DOI:** 10.1128/mbio.01822-25

**Published:** 2025-09-08

**Authors:** Anita Tarasenko, Bhavya N. Papudeshi, Susanna R. Grigson, Vijini Mallawaarachchi, Abbey L. K. Hutton, Morgyn S. Warner, Jeremy J. Barr, Jon Iredell, Bart Eijkelkamp, Robert A. Edwards

**Affiliations:** 1 Flinders Accelerator for Microbiome Exploration, College of Science and Engineering, Flinders University 1065, Adelaide, South Australia, Australia; 2 Microbiology & Infectious Diseases Directorate, SA Pathology, Central Adelaide Local Health Network 375072, Adelaide, South Australia, Australia; 3 Faculty of Health and Medical Sciences, University of Adelaide 1066, Adelaide, South Australia, Australia; 4 School of Biological Sciences, Monash University 2541, Melbourne, Victoria, Australia; 5 Centre for Infectious Diseases and Microbiology, The Westmead Institute for Medical Research, Westmead, New South Wales, Australia; 6 Faculty of Medicine and Health, University of Sydney 4334, , Sydney, New South Wales, Australia; 7 Western Sydney Local Health District, Westmead Hospital 8539, Westmead, New South Wales, Australia; 8 Sydney Institute for Infectious Diseases, The University of Sydney 4334, Sydney, New South Wales, Australia; 9 College of Science and Engineering, Flinders University 1065, Bedford Park, South Australia, Australia; Instituto Carlos Chagas, Curitiba, Brazil

**Keywords:** multidrug resistance (MDR), ESKAPEE pathogens, phage therapy, efflux pump inhibition, phage-antibiotic synergy

## Abstract

Multidrug-resistant (MDR) and extensively drug-resistant (XDR) ESKAPE pathogens pose a significant global health threat due to their ability to evade antibiotics through intrinsic and acquired mechanisms. These bacteria, including *Enterococcus faecium*, *Staphylococcus aureus*, *Klebsiella pneumoniae*, *Acinetobacter baumannii*, *Pseudomonas aeruginosa*, *Escherichia coli,* and *Enterobacter* species, evade antibiotics through intrinsic and adaptive mechanisms. Common strategies include capsule formation, biofilm, β-lactamase production, and efflux activity. Using these mechanisms, bacteria can evade the effects of antibiotics, leading to persistent and difficult-to-treat infections. Understanding the mechanisms of resistance is crucial in developing effective strategies to combat MDR and XDR ESKAPEE pathogens. A promising approach is the development of alternative treatments targeting specific resistance mechanisms in these pathogens. Bacteriophages (phages), which co-evolve with bacterial hosts, offer a dynamic therapeutic alternative by targeting pathogenic bacteria using precision-based strategies. This targeted approach can overcome antibiotic resistance and reduce the risk of damaging the beneficial microbiota. Phages can restore susceptibility in previously untreatable infections by enhancing antibiotic uptake and imposing fitness costs on resistant strains. However, therapeutic deployment faces challenges such as rapid evolution of phage resistance, inconsistent production standards, and limited regulatory pathways. This review examines the mechanistic insights into phage-antibiotic synergy, with a focus on efflux pump-mediated resistance. It discusses emerging therapeutic strategies, current clinical applications, and the translational frameworks needed to integrate phage therapy into mainstream medicine and transform the clinical management of drug-resistant ESKAPEE infections.

## INTRODUCTION

Antimicrobial resistance (AMR) is one of the greatest threats to global health. If unaddressed, it is projected to cause over 39 million cumulative deaths globally by 2050 (1). While resistance is a broad and evolving problem, a small group of bacteria, collectively known as ESKAPEE pathogens, has emerged as particularly concerning contributors due to their high prevalence in healthcare settings and extensive resistance (2, 3). These bacteria, comprising *Enterococcus faecium, Staphylococcus aureus, Klebsiella pneumoniae, Acinetobacter baumannii, Pseudomonas aeruginosa, Enterobacter* spp*.,* and *Escherichia coli* (4), are MDR and XDR strains that defy current treatment options.

The alarming decline in effective antibiotic options, particularly the emergence of resistance to last-resort treatments, carbapenems and vancomycin, has renewed interest in phage therapy as an alternative approach. Bacteriophages (phages), viruses that infect and lyse bacteria, offer a targeted treatment approach that can circumvent conventional antibiotic resistance mechanisms (5). Phages achieve this by exploiting essential bacterial surface structures, such as outer membrane proteins, lipopolysaccharides, capsule polysaccharides, or efflux components, to recognize and bind to specific bacterial receptors. This specificity not only enables targeted lysis of bacteria but also minimizes damage to the host microbiome, in contrast to broad-spectrum antibiotics (6). Against this mechanistic background, recent clinical applications of phage therapy have shown promise in treating otherwise intractable infections, highlighting its potential as a viable complement or alternative to traditional antibiotics.

The recent successful clinical use of phages and antibiotics to treat infections in patients with serious or life-threatening conditions demonstrates the therapeutic value against MDR and XDR pathogens (7). A growing body of case studies and clinical trials provides compelling evidence that phages, primarily when used in combination with antibiotics, can be effective against MDR and XDR infections caused by ESKAPEE pathogens (Table 1).

**TABLE 1:** Clinical and compassionate use of phage therapy against ESKAPEE pathogens with potential efflux pump involvement

Pathogen	Phage/antibiotic used	Targeted efflux pump	Antibiotics used	Outcome/success rate	Use type	Reference(s)
*Acinetobacter baumannii*	Phage cocktail (including ΦAbN1, Bϕ-Ab02, and AB-P-01), IV and nebulized. Inhaled ΦAbN1 only (in a separate case)	*adeABC* (suspected) *adeB* (engineered phage disruption *in vitro*)	Meropenem, colistin, ceftazidime-avibactam (varied)	3/3 cases resolved infection (100%). One patient with XDR pneumonia, one with bloodstream infection, one with osteomyelitis	Compassionate	(8–10)
*Klebsiella pneumoniae*	ΦKpNIH1 and ΦKpNIH2, orally + rectally. Other cocktails in five total patients	Unknown (efflux contribution likely in KPC strains)	Meropenem, colistin, ceftazidime-avibactam (varied)	4/5 cases were successful (80%). A patient died from unrelated sepsis. Stool colonization also reduced in 3 cases.	Compassionate	(11)
*Pseudomonas aeruginosa*	Pae103, Pae201 (nebulized) phage PaP1, PP1131, and others	*MexAB-OprM*, *MexXY-OprM* (suspected/targeted *in vitro*)	Tobramycin, ceftazidime-avibactam, piperacillin-tazobactam	~70–80% success rate across multiple CF and ventilator-associated cases. Observed synergy with phages restoring drug susceptibility	Compassionate + clinical trial	(12, 13)
*Staphylococcus aureus*	AB-SA01 (Myoviridae cocktail), IV	Not applicable (Gram-positive)	Vancomycin, daptomycin, flucloxacillin (varied)	13/15 patients in the trial showed clinical improvement or pathogen clearance (87%)	Phase 1 clinical trial	(14)
*Enterococcus faecium*	Not disclosed (cocktail of 2–3 phages), oral and rectal	Efflux role unclear	Daptomycin, linezolid, tigecycline	2/3 cases showed prolonged symptom-free periods, but eventually required re-treatment	Compassionate	(15, 16)
*Escherichia coli*	Custom phage + meropenem (urosepsis) ΦES17 (engineered for UTI) Other case series	*AcrAB-TolC* (*in vitro* targeting shown)	Meropenem, ciprofloxacin, nitrofurantoin	~75–80% resolution in individual case reports and small series. Engineering phage to degrade TolC restored antibiotic susceptibility	Compassionate + experimental	(17)
*Enterobacter cloacae*	Lytic phage cocktail + AI-based dosing optimization (*in silico* + clinical)	Unknown (efflux likely in XDR strain)	Meropenem, ceftazidime-avibactam	Infection cleared in a compassionate case; predicted enhanced outcomes with optimized dosing.	Compassionate + model	(13)

Despite these promising outcomes, several challenges remain. These include the rapid evolution of phage resistance, regulatory hurdles delaying approval, and the need for scalable and reproducible production pipelines. More fundamentally, there remains a limited understanding of the underlying resistance mechanisms that drive both antibiotic failure and phage evasion, as well as how phage-antibiotic synergy operates to overcome these barriers.

Recent clinical insights emphasize that successful phage therapy depends on a mechanistic understanding of both bacterial resistance and phage infectivity. Evidence suggests that personalized approaches, tailoring phages to the specific efflux-dominant bacterial strains in individual patients, are crucial for ensuring therapeutic efficacy (18). Moreover, the dynamic nature of phage–bacterium interactions often necessitates adaptable treatment regimens, including reformulated phage cocktails to counter emerging resistance (19). Host-related factors such as immune status, infection site, and the presence of biofilms further influence outcomes.

These clinical realities emphasize the value of mechanistic research, such as understanding phage–efflux pump interactions, not only to predict and enhance therapeutic efficacy but also to inform real-time susceptibility testing and dynamic treatment design. As the field progresses toward broader clinical implementation, understanding these mechanisms will be essential for developing precision medicine strategies for efflux-driven MDR infections (20).

This review outlines the resistance mechanisms that define ESKAPEE pathogens and explores how phage–antibiotic synergy, particularly through the targeting of efflux systems, can dismantle MDR phenotypes and enhance treatment outcomes.

## EFFLUX PUMPS AS MULTIDIMENSIONAL DRIVERS OF PATHOGENICITY

Efflux pumps are increasingly recognized as central contributors to both the antimicrobial resistance and pathogenic potential of ESKAPEE pathogens. Initially characterized for their role in exporting antibiotics and toxic metabolites, these membrane-embedded transporters have emerged as versatile systems that influence a range of bacterial phenotypes, including virulence, biofilm formation, and environmental adaptation. By enabling survival under pharmacological and physiological stress, efflux pumps represent dual-purpose targets for both antimicrobial and antivirulence interventions.

### Structural and functional overview

**Fig 1:**
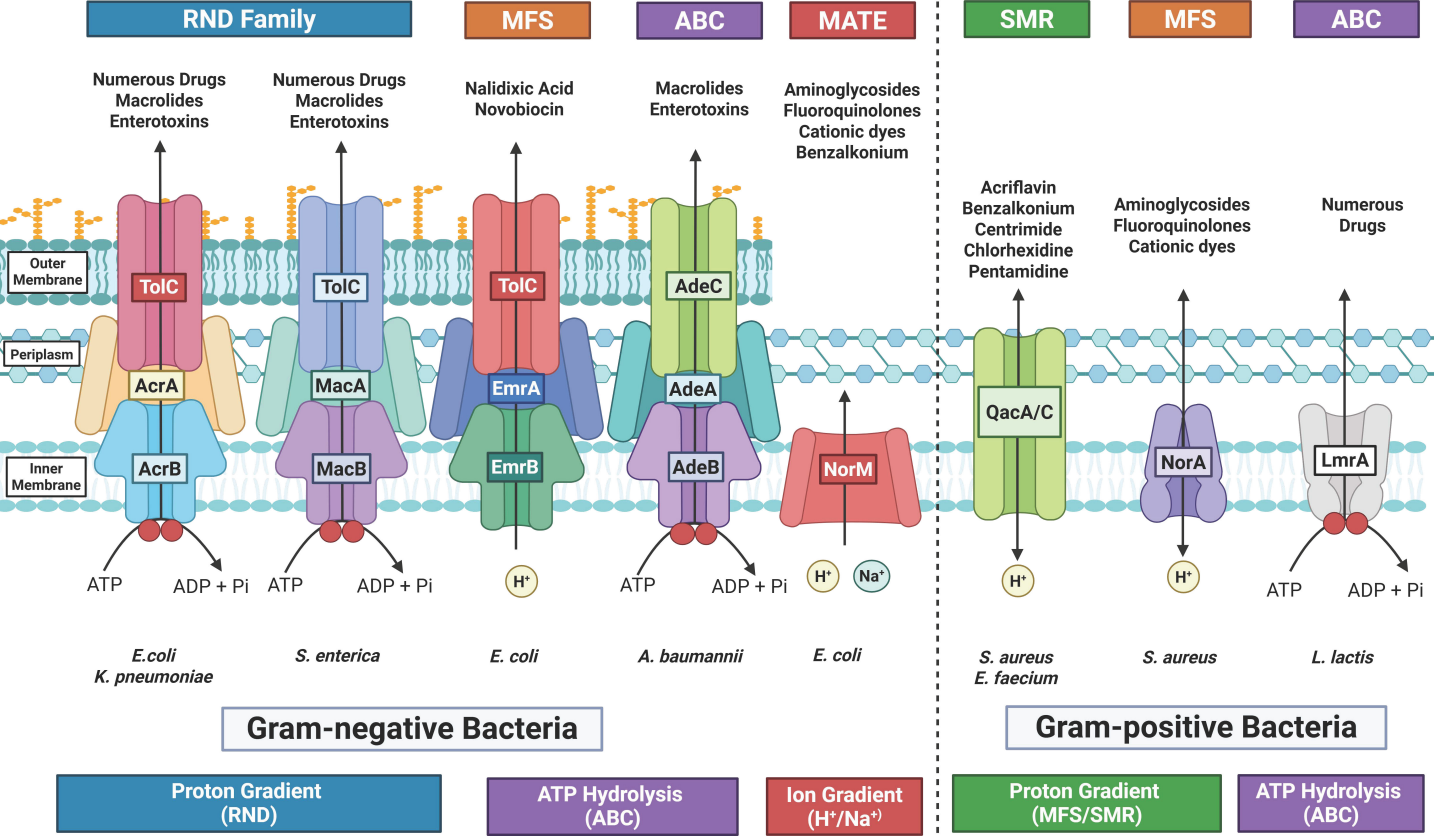
Schematic of major efflux pump families in Gram-negative and Gram-positive bacteria, highlighting structural organization, energy sources, representative transporters, and known substrates. MATE transporters (e.g., NorM) use H^+^ or Na^+^ gradients, contrasting with ATP-driven ABC systems or proton-driven RND, MFS, and SMR pumps. Drug classes exported include fluoroquinolones, macrolides, aminoglycosides, and antiseptics (created with BioRender.com/t85dj13, accessed 5 May 2025).

Efflux pumps are broadly categorized into five principal families based on structure, energy source, and substrate specificity. Among these, the Resistance-Nodulation-Division (RND) family is the most clinically relevant in Gram-negative bacteria, particularly in *A. baumannii, E. coli, K. pneumoniae,* and *P. aeruginosa* (21–23). These systems form tripartite complexes that span the inner membrane, periplasm, and outer membrane, allowing direct expulsion of toxic compounds into the environment (Fig. 1). The RND family includes AcrAB-TolC in *E. coli* and *K. pneumoniae*, MexAB-OprM in *P. aeruginosa*, and AdeABC in *A. baumannii* (21–23). These systems are proton motive force-driven and capable of exporting β-lactams, quinolones, and tetracyclines.

The major facilitator superfamily (MFS) is widely distributed across both Gram-positive and Gram-negative bacteria and uses the proton gradient to export drugs. For example, NorA in *S. aureus* confers fluoroquinolone resistance, while EmrAB in *E. coli* contributes to multidrug efflux (Fig. 1) (24).

The multi-antimicrobial extrusion (MATE) family is also widespread across bacterial species, but uses sodium ion gradients for transport. For example, MepA in *S. aureus* expels cationic antimicrobials and fluoroquinolones (Fig. 1) (25).

The small multidrug resistance (SMR) transporters are compact systems often conferring low-level resistance, particularly in environmental and nosocomial strains (Fig. 1) (26).

Finally, ATP-binding cassette (ABC) transporters like LmrA in *Lactococcus* spp. and EfrAB in *Enterococcus* spp. harness ATP hydrolysis to drive efflux (Fig. 1) (27).

What makes these systems particularly formidable is their broad substrate specificity; a single pump can export structurally unrelated compounds and their energetic flexibility, utilizing either ion gradients (RND, MFS, MATE) or ATP (ABC) as a power source. This adaptability allows bacteria to respond to adverse environmental conditions, including antibiotic pressure.

### Regulation and environmental induction

Efflux pump expression is typically under tight regulatory control to avoid the fitness cost of constitutive expression. However, in resistant clinical isolates, mutations in both local and global regulatory systems often result in derepression or overactivation. Local repressor genes such as *acrR* (*E. coli*), *mexR* (*P. aeruginosa*), and *adeN* (*A. baumannii*) function as negative regulators of efflux operons (28, 29). Loss-of-function mutations in these repressors lead to uncontrolled pump expression. Activation of global stress-response regulators like *marA, soxS,* and *rob* orchestrates the expression of multiple efflux systems, as well as stress response genes. These regulators are often upregulated under oxidative stress, antibiotic exposure, and envelope perturbation.

For example, sub-inhibitory concentrations of ciprofloxacin can induce *marA* and *soxS,* triggering efflux upregulation and increased mutation rates (30, 31). Similarly, *adeABC,* in *A. baumannii,* is inducible by chloramphenicol and ethanol, illustrating the environmental sensitivity of these systems (32). This context-dependent inducibility allows pathogens to rapidly shift into a drug-resistant phenotype upon antibiotic exposure, contributing to treatment failure and persistent infections.

### Role of antimicrobial resistance

Efflux pumps are key mediators of intrinsic and acquired resistance across the ESKAPEE pathogens. By actively exporting antibiotics before they reach their intracellular targets, these systems can lower intracellular drug concentrations below the minimum inhibitory concentration (MIC), rendering therapy ineffective, even in the absence of target mutations or enzymatic degradation. Quantitative epidemiological data highlight their clinical relevance. In carbapenem-resistant *A. baumannii*, overexpression of the *adeABC* operon is observed in approximately 70% of isolates, particularly in ICUs across Southeast Asia and Latin America (33, 34). In fluoroquinolone-resistant *K. pneumoniae, acrAB-TolC* overexpression is reported in >50% of clinical isolates in India, southern Europe, and China (35).

The impact of efflux is not only qualitative but also quantitative. The overexpression of the AcrAB-TolC efflux pump can increase MICs for tetracycline and chloramphenicol by up to 64-fold. By contrast, the MexAB-OprM efflux pump in *P. aeruginosa* can elevate resistance to β-lactams and macrolides by 8- to 32-fold (36, 37).

These values highlight efflux as a convergent resistance mechanism, capable of simultaneously conferring resistance to multiple, unrelated antibiotic classes via a single transport system. This breadth of activity makes efflux pumps both formidable resistance determinants and ideal therapeutic targets.

### Role of virulence and biofilm formation

Beyond antimicrobial resistance, efflux pumps contribute significantly to pathogenesis and persistence in host environments. Their role extends to quorum sensing, biofilm formation, toxin secretion, and metabolic adaptation. In *P. aeruginosa,* the MexAB-OprM system is essential for quorum sensing, mediating the secretion of acyl-homoserine lactones such as 3-oxo-C12-HSL, which regulate population-level behaviors including virulence gene expression and biofilm formation (38). These pumps also contribute to biofilm matrix production by exporting extracellular polymeric substances that form the structural scaffold of biofilms, thereby promoting immune evasion and antibiotic toleration. Inhibition of MexAB-OprM disrupts biofilm architecture and diminishes the bacterium’s colonization capacity. In *E. coli*, the AcrAB-TolC system facilitates the export of hemolysin and other cytotoxic factors, enhancing tissue damage and evasion of host immune response (39). Similarly, in *A. baumannii*, the AdeABC efflux pump supports metabolic adaptability and resistance to oxidative stress, enabling survival in hostile environments such as disinfected surfaces and bloodstream niches (40). Collectively, these systems emphasize the multifaceted contributions of efflux pumps to bacterial virulence, persistence, and environmental resilience.

These pleiotropic roles suggest that targeting efflux systems may yield dual benefits: reversing drug resistance and attenuating virulence. This dual functionality positions efflux pumps as high-value targets for multi-modal therapeutic strategies, particularly those that combine conventional antibiotics with efflux-disrupting agents such as phages.

## PHAGES: A POTENTIAL SOLUTION

Building on the multifaceted role of efflux pumps in both resistance and virulence, bacteriophage (phage) therapy offers a promising strategy to exploit this vulnerability. Phages specifically target bacterial surface structures, including efflux pump components, making them uniquely suited to undermine multidrug resistance and pathogenicity simultaneously. By selectively infecting and lysing efflux-active cells, phages can dismantle bacterial defenses from the outside in, disrupting both drug efflux and virulence pathways in the process.

### Advantages of phage therapy over traditional antibiotics

Phages possess several advantages over conventional antibiotics, particularly in the context of MDR and XDR infections, where efflux-mediated resistance is prevalent (5). Unlike antibiotics, which are often broad-spectrum and microbiota-disruptive, phages exhibit strain-level specificity. This precision reduces the risk of dysbiosis and secondary infections and may preserve commensal bacteria that contribute to colonization resistance.

A key advantage of phages is their evolutionary adaptability. In contrast to static antibiotic molecules, phages co-evolve with their bacterial hosts, overcoming resistance through mechanisms such as receptor mutations, CRISPR-Cas defense systems, and abortive infection pathways (41). This dynamic arms race enables phages to rapidly adapt through mutations, recombination, or gene acquisition, thereby overcoming bacterial defenses and rendering antibiotics ineffective. When bacteria acquire resistance to phage infection, often through mutations or downregulation of surface receptors such as TolC or OprM, they frequently incur functional trade-offs (42). These include impaired efflux capacity, altered membrane permeability, or reduced biofilm formation, all of which increase susceptibility to antibiotics (43).

Moreover, phages also replicate *in situ*, naturally replicating at the site of infection. If susceptible bacteria are present, phages replicate, ensuring a self-limiting and localized increase in therapeutic concentration (44). By contrast, antibiotics typically require repeated high doses to maintain therapeutic levels, which can increase the risk of systemic toxicity and accelerate the emergence of resistant strains. Despite these advantages, the translational integration of phage therapy remains limited by infrastructural and regulatory barriers. Challenges include the need for rapid, patient-specific phage matching, batch consistency, endotoxin removal, and validated susceptibility testing (20). However, recent developments in synthetic biology, curated phage banks, and adaptive regulatory models are beginning to address these limitations.

Understanding the mechanistic interactions between phages, bacterial resistance systems, and host adaptation is crucial for developing targeted therapies. By optimizing phage infection mechanisms and combining lytic activity with genetic or regulatory suppression, we can resensitize MDR and XDR pathogens to antibiotics.

### Mechanistic diversity and therapeutic potential of phages

Phages display diverse infection strategies beyond the classical lytic, temperate, pseudolysogenic, and chronic infection modes, each with therapeutic implications (45). Lytic phages hijack the bacterial transcriptional and translational machinery, ultimately causing cell lysis and bacterial death, traits that make them ideal for their bactericidal application and minimize the risk of long-term genomic integration (46).

By contrast, temperate phages integrate into the host genome as prophages and may carry accessory genes, including those encoding toxins or efflux pump regulators (47). While temperate phages pose risks such as horizontal gene transfer and virulence enhancement, advances in genome editing have enabled their conversion to an obligately lytic form. Yet, they can modulate bacterial physiology and resistance in beneficial ways (48). Lysogeny and pseudolysogeny enable phages to remain dormant in metabolically inactive cells. By contrast, chronic infections involve continuous phage replication without host cell lysis, resulting in gradual alterations to the host’s function.

Importantly, phage resistance frequently comes at a cost (49). When bacteria alter or downregulate essential surface receptors, such as efflux pump components, to evade phage attack, they often sacrifice critical resistance functions (50, 51). For example, deletion or mutation of TolC (*E. coli*) or OprM (*P. aeruginosa*) impairs the function of the RND efflux complexes, reducing resistance to β-lactams, fluoroquinolones, and macrolides (52, 53). In this way, even incomplete phage clearance can resensitize bacteria to conventional antibiotics by forcing them into a vulnerable phenotype.

These dynamics suggest that phage-antibiotic synergy can be engineered intentionally. By selecting phages that target structurally or functionally essential components of efflux systems, treatment can drive bacterial evolution toward susceptibility, making the surviving population more treatable with existing drugs.

### Structural determinants and therapeutic targeting of phage receptors in ESKAPEE pathogens

Phage infection begins with the recognition and binding to specific bacterial surface receptors, initiating DNA injection. In ESKAPEE pathogens, these receptors are often integral components of resistance and virulence systems, offering high-value targets for therapeutic intervention. Examples include capsular polysaccharide, outer membrane proteins such as OmpA in *A. baumannii*, TolC in *E. coli* and *K. pneumoniae*, OprM in *P. aeruginosa*, as well as components of multidrug efflux pumps (54–57). Many phages, particularly those infecting Gram-positive bacteria, use a two-step attachment process: initial reversible adherence to ubiquitous surface structures, such as LPS or teichoic acids, followed by irreversible docking onto specific high-affinity receptors, including efflux-associated proteins or capsule components (58, 59).

By targeting essential structures, phages exploit a key bacterial vulnerability: these receptors are conserved under selective pressure because altering them carries a fitness cost. For instance, mutation of TolC can prevent phage entry but also inactivate the AcrAB-TolC efflux system, increasing antibiotic sensitivity and reducing virulence. Similarly, phages targeting OprM in *P. aeruginosa* can select for mutants with impaired biofilm formation and increased drug susceptibility. To overcome protective barriers, such as bacterial capsules, many phages encode polysaccharide depolymerases. These enzymes degrade the extracellular matrix, exposing buried receptors and facilitating access to efflux pump components embedded in the outer membrane (54, 60, 61). In addition to improving phage infectivity, depolymerases can disrupt biofilm integrity and enhance the penetration of antibiotics and immune factors (62).

The therapeutic implications are significant: by targeting essential efflux-associated receptors, phages force bacteria into evolutionary trade-offs where resistance to phages often comes at the cost of reduced antibiotic resistance or virulence. Mutations that block phage binding usually compromise the function of efflux systems, resulting in increased antibiotic susceptibility, reduced virulence, and impaired membrane integrity (63–65). These trade-offs reshape the bacterial population toward treatment sensitivity, especially under combined phage-antibiotic pressure.

This phage-antibiotic synergy is further amplified through co-administration strategies. Phages selectively lyse efflux-active cells, while efflux pump inhibitors (EPIs) block residual activity in bacteria that survive. As efflux is suppressed, antibiotics accumulate intracellularly and exert their full bacterial effect (65, 66). Together, these combinations of phage, EPI, and antibiotics form a triple-threat intervention that not only enhances bacterial killing and delays the emergence of resistance subpopulations more effectively than any monotherapy, but also shows particular promise against efflux-dominant pathogens where pump overexpression is chromosomally encoded and conserved across clinical isolates.

## PHAGE-ANTIBIOTIC SYNERGY TARGETING EFFLUX SYSTEMS

Building on the foundational role of efflux pumps in antimicrobial resistance and virulence, and their emerging vulnerabilities to phage infection, the section explores how efflux systems can be exploited as dual-purpose therapeutic targets. By combining phages with antibiotics or EPIs, treatment regimens can simultaneously disrupt bacterial survival mechanisms, restore antibiotic efficacy, and delay the emergence of resistance. This section examines efflux architecture, regulation, vulnerability to phage binding, and clinical application of efflux-targeted phage-antibiotic synergy.

### Efflux pump induction in response to antibiotic pressure

Efflux pump overexpression is a key adaptive mechanism in bacteria that respond to antibiotic pressure. Under normal conditions, transcription of efflux operons is tightly repressed, such as AcrR (*E. coli*), MexR (*P. aeruginosa*), and AdeN (*A. baumannii*) (67–70). However, spontaneous, antibiotic-inducible, or horizontally acquired mutations in these repressors lead to derepression and constitutive pump expression, significantly enhancing multidrug resistance (71).

In addition to local regulators, global transcriptional activators such as MarA, SoxS, and Rob respond to envelope stress, oxidative damage, and sub-inhibitory antibiotic exposure by coordinating the expression of multiple efflux systems and stress response genes (72). For example, sub-MIC levels of ciprofloxacin upregulate *marA* and *soxS*, leading to increased efflux and a rise in mutation rates (73). In *A. baumannii*, AdeABC is inducible by environmental cues like ethanol and chloramphenicol, highlighting the contextual sensitivity of efflux regulation (32).

At a broader regulatory level, global repressors like the histone-like nucleoid structuring protein (H-NS) modulate the expression of horizontally acquired genes, including resistance loci. Inactivation of H-NS has been shown to derepress multiple efflux operons, enhancing survival under antimicrobial stress while complicating efforts to suppress resistance uniformly (74). This multi-layered regulation, spanning operon-specific repressors to global chromosomal regulators, enabled targeted and systematic induction of efflux activity in response to antibiotics.

Importantly, efflux induction has pleiotropic consequences beyond antibiotic export. Overexpression of AcrAB-TolC in *E. coli* alters membrane permeability, oxidative stress tolerance, and cellular metabolism. Similar effects are observed in *P. aeruginosa* and *S. aureus*, where elevated efflux correlates with increased persistence and survival under host-imposed stresses (75). Efflux activity also interacts with other resistance mechanisms, such as β-lactamase production, porin loss, and target-site modification, to create multidimensional resistance profiles, particularly in carbapenem-resistant Gram-negative bacteria and macrolide- or fluoroquinolone-resistant Gram-positive species (76, 77).

However, this regulatory plasticity comes with a therapeutic trade-off. Many efflux pumps, especially RND-type systems like TolC and OprM, are not only essential for resistance but also serve as phage receptors. Antibiotic-driven overexpression of these components can inadvertently increase phage susceptibility. Conversely, bacterial strategies to evade phage predation, such as receptor downregulation or mutation, frequently impair efflux function and resensitize bacteria to antibiotics.

This evolutionary tension, between resistance via efflux and vulnerability via phage targeting, creates a unique opportunity for combination therapies. By harnessing phages that exploit overexpressed efflux components, treatment can steer bacterial populations toward fitness-compromising mutations, reversing resistance and restoring antibiotic efficacy. This concept is developed further in the sections that follow.

### Efflux system as therapeutic entry points

Efflux pumps are critical to bacterial resistance and virulence in ESKAPEE pathogens, but paradoxically, they also expose bacteria to phage-mediated attack. Several components of these systems, particularly outer membrane proteins, serve as receptors for lytic phages, converting key resistance determinants into potential therapeutic targets.

This structural vulnerability is especially evident in Gram-negative bacteria, where surface-exposed components of RND-type efflux pumps double as phage receptors. For instance, TolC in *E. coli* and *K. pneumoniae* is exploited by phages U136B and phi92, while OprM, part of the MexAB-OprM system in *P. aeruginosa*, is targeted by PAK_P1 (78–80). In *A. baumannii*, the multifunctional outer membrane protein OmpA, involved in both adhesion and efflux, is bound by phage AB1 (81, 82). These examples reveal a recurring pattern across ESKAPEE species: essential, surface-accessible efflux components are often co-opted by phages as entry points.

The therapeutic implications of this receptor duality are profound. When phages use efflux machinery for adsorption, they impose selective pressure that forces bacteria to either retain these receptors and remain phage susceptible or modify them at the cost of efflux function. Mutations in TolC, for example, may prevent phage binding but disrupt the AcrAB-TolC complex, reducing antibiotic efflux and re-sensitizing *E. coli* to β-lactams and fluoroquinolones (78). Likewise, loss or modification of OprM impairs biofilm formation, quorum sensing, and resistance to macrolides and aminoglycosides in *P. aeruginosa* (83). Similar vulnerabilities have been documented for OmpA in *A. baumannii*, underscoring the broad relevance of this mechanism.

These evolutionary trade-offs have been validated in both *in vitro* and *in vivo* systems. Exposure to efflux-targeting phages consistently leads to the emergence of receptor-deficient mutants with increased antibiotic sensitivity (78). For example, in attempting to evade PAK_P1 infection, *P. aeruginosa* mutants that lose OprM often compromise efflux function, resulting in heightened susceptibility to macrolides and aminoglycosides. Similarly, *E. coli* or *K. pneumoniae* strains that lose TolC under phage pressure become resensitized to β-lactams and fluoroquinolones. In some cases, minimum inhibitory concentrations (MICs) are reduced by up to 16-fold, even in the absence of efflux pump inhibitors (78, 84).

What makes this strategy particularly effective is the functional constraint imposed by the efflux systems themselves. Proteins like TolC and OprM are deeply embedded in bacterial resistance and fitness networks, and their conservation across clinical isolates makes them poor targets for mutational evasion. Phages that bind these receptors exploit this evolutionary rigidity, creating a therapeutic bottleneck that bacteria cannot easily circumvent.

By leveraging these dynamics, phage therapy redefines efflux systems not only as contributors to resistance but as entry points for therapeutic disruption. Rather than bypassing efflux-mediated resistance, this approach directly engages it, selectively undermining a core bacterial survival mechanism while restoring antibiotic efficacy.

### Engineered phages and CRISPRi-enhanced strategies

While naturally occurring phages offer powerful tools for targeting efflux-dominant bacteria, advances in synthetic biology and genetic engineering have enabled the development of engineered phages that go beyond lysis to deliver precise molecular payloads. These next-generation phages can be programmed to suppress resistance mechanisms at the transcriptional or post-transcriptional level, without requiring bacterial lysis, thereby enhancing synergy with antibiotics and expanding therapeutic flexibility (17).

One promising approach involves the delivery of CRISPR interference (CRISPRi) systems via engineered phages. CRISPRi uses a catalytically inactive Cas9 (dCas9) protein, which is guided by custom-designed single guide RNAs (sgRNAs) to bind specific DNA sequences, thereby blocking transcription without introducing double-stranded breaks. This mechanism is ideal for downregulating essential but non-lethal targets, such as efflux pump operons, where outright knockout may select for escape mutants or cause unintended stress responses (85).

In parallel, phages can also be engineered to express antisense RNAs, which hybridize to target mRNA transcripts and inhibit translation, effectively silencing gene expression post-transcriptionally. This approach has been beneficial when precise temporal control or transient suppression of gene activity is needed, such as during antibiotic co-treatment windows (86).

These strategies have shown promising results in preclinical studies. For instance, engineered CRISPRi-phages targeting the *acrAB* operon in *E. coli* have led to up to 8-fold reductions in ciprofloxacin MICs, significantly enhancing bactericidal activity even in cells that evade initial lysis. Similar decreases in resistance have been achieved in *P. aeruginosa* and *A. baumannii* by silencing *mexAB* and *adeABC*, respectively, demonstrating the broad applicability of these systems across high-priority ESKAPEE pathogens (85).

What makes these tools particularly impactful is their relevance in efflux-dominant strains, where pump overexpression is often chromosomally encoded, conserved across clinical isolates, and difficult to eliminate through conventional methods. Traditional inhibitors or resistance gene knockouts may be ineffective or rapidly countered by compensatory mechanisms. By contrast, CRISPRi and antisense phages allow for targeted, finely controlled suppression of efflux activity, minimizing off-target effects and preserving the viability of non-target microbiota (17, 86).

Moreover, because engineered phages retain their host specificity and replication capacity, these interventions can be self-amplifying, spreading gene-silencing activity through the bacterial population during an infection. This property not only improves efficacy but also reduces the dosing burden and systemic toxicity compared to traditional combination therapies (17).

In sum, engineered phages equipped with genetic silencers represent a new class of programmable therapeutics. By selectively disabling efflux systems from within, they can reopen previously closed therapeutic windows, enhance antibiotic potency, and prevent resistance rebound, all while exploiting the natural dynamics of phage-host interaction. As these technologies mature, they may enable customized, pathogen-specific interventions that adapt in real time to the molecular signature of each infection (17).

### Multi-pronged therapeutic combinations targeting efflux

Efflux pump overexpression is a major driver of multidrug resistance in ESKAPEE pathogens and poses a formidable barrier to antibiotic efficacy (87). Overcoming this requires a multi-faceted approach that targets the bacterial cell from multiple angles. Combining bacteriophages with antibiotics and EPIs creates a synergistic, layered attack that disrupts efflux-mediated defenses through complementary mechanisms (51).

In this strategy, phages selectively infect and lyse efflux-active cells by exploiting outer membrane components such as TolC, OprM, or OmpA. These structures are often overexpressed and structurally constrained in resistant strains (88). Meanwhile, EPIs such as phenylalanine-arginine β-naphthylamide or carbonyl cyanide m-chlorophenyl hydrazone act intracellularly to block residual efflux pump activity in surviving cells, preventing the export of antibiotics. This suppression allows conventional antibiotics, previously rendered ineffective, to accumulate intracellularly at bactericidal concentrations and resume their intended function (89).

Together, this combination achieves therapeutic outcomes that are rarely possible with single or dual agents. It not only enhances bacterial killing but also delays or prevents the emergence of resistant subpopulations (64). *In vitro* studies have shown that when phages targeting TolC or OprM are combined with EPIs and antibiotics such as ciprofloxacin, they produce complete eradication of resistant bacteria and prevent rebound growth, even in challenging conditions such as high-density biofilms. These effects are exceptionally robust in clinical isolates where efflux pump overexpression is chromosomally encoded and thus highly conserved. In such strains, mutational escape from phage targeting or efflux inhibition is often constrained by the fitness cost of altering essential membrane components or losing efflux functionality altogether.

The therapeutic advantages of this approach are manifold. It reduces the risk of selecting for resistance to any one component, broadens the efficacy of existing antibiotics without needing to develop new ones, and allows the reintroduction of drugs previously sidelined due to efflux-mediated resistance. Moreover, because phages are highly specific to their bacterial targets, this strategy limits off-target effects and minimizes disruption of the commensal microbiota. By transforming efflux systems from protective barriers into structural liabilities, this triple-combination approach exemplifies a shift in antimicrobial strategy, from simply trying to overpower resistance to re-engineering the evolutionary pressures that sustain it (90). In doing so, it provides a robust and adaptable framework for addressing infections that have become refractory to conventional treatment.

## INNOVATIONS AND FUTURE HORIZONS

As global antibiotic resistance accelerates, phage therapy must evolve from a niche experimental concept to a credible clinical intervention. Its future depends not only on discovering new natural phages, but also on intentionally engineering precision-based, resistance-aware therapeutics. This next generation of phage therapies will focus on selectively disrupting key resistance mechanisms, especially efflux pumps, through structural targeting, exploitation of evolutionary trade-off exploitation, and adaptive combination strategies.

One of the most transformative advances supporting this future is the rise of protein structure prediction technologies, such as AlphaFold, which now enable accurate modeling of phage receptor-binding proteins at atomic resolution (91–93). These tools have revolutionized our ability to rationally design or modify phages to retain infectivity even against bacterial strains with mutated or structurally altered surface proteins like TolC, OprM, or OmpA (94). Phages can now be engineered to recognize variant receptor topologies or to carry depolymerase enzymes that degrade capsules and biofilms, thereby exposing buried efflux components and improving therapeutic access. These molecular refinements increase phage penetration, expand host range, and deepen synergy with antibiotics, particularly in biofilm-dense or capsulated infections.

Beyond surface targeting, engineered phages equipped with gene-silencing tools, such as CRISPR interference (CRISPRi) systems and antisense RNAs (14). These phages go beyond lysis: they can inhibit efflux pump expression from within, silencing genes like *acrAB*, *mexAB*, or *adeABC*. CRISPRi-phages use a catalytically inactive Cas9 (dCas9) protein to block transcription, while antisense RNAs bind mRNA to prevent translation (95). Both approaches increase intracellular antibiotic concentrations without killing the bacteria outright, weakening resistance and improving outcomes when paired with traditional antimicrobials (96).

These engineered systems are particularly advantageous in efflux-dominant pathogens, where overexpression is chromosomally encoded and conserved across isolates. Traditional inhibitors often fail in such cases due to compensatory mutations or off-target toxicity. Engineered phages, however, can be reprogrammed dynamically, evade bacterial immune defenses such as CRISPR-Cas or restriction-modification systems, and adapt to new resistance profiles. Because they retain natural replication ability, they also amplify their therapeutic effects at the site of infection, reducing systemic dosing requirements and minimizing toxicity.

To translate these innovations into practice, robust clinical pipelines and regulatory models are needed. Manufacturing must meet good manufacturing practice (GMP) standards, with consistent phage quality, minimal endotoxin levels, and validated host range data (97). Regulatory pathways must also evolve to accommodate phage-specific characteristics, such as the need for personalized matching, iterative formulation updates, and dynamic susceptibility testing (98).

Looking forward, multi-modal therapeutic models will define the next chapter in antimicrobial strategy. Phage–antibiotic–EPI tri-therapies are especially promising, as they disable efflux function from multiple directions, restoring the efficacy of previously obsolete antibiotics (51, 99). These combinations also allow for lower antibiotic dosages, preserving microbiota integrity and reducing toxicity. In parallel, phages that degrade capsules or biofilms inadvertently enhance susceptibility to host immunity, including neutrophil clearance and complement activation (100, 101). This crosstalk between phage therapy and host defenses offers a fourth layer of synergy that is just beginning to be explored.

The future phage therapy will not rely solely on natural isolates, but rather on a new class of programmable, resistance-aware biologics designed to adapt to the shifting landscape of antimicrobial resistance. Through structural redesign, receptor-target mapping, evolutionary manipulation, and engineered gene suppression, phages will serve not only as direct antimicrobials but also as vehicles for molecular reprogramming of bacterial behavior. As these tools evolve, they will enable precision eradication of resistant infections and redefine the clinical management of MDR and XDR pathogens.

## CONCLUSION

Efflux pumps remain a central player in multidrug resistance in ESKAPEE pathogens, undermining antibiotic efficacy and limiting treatment options. However, their structural conservation, functional indispensability, and dual role as phage receptors create a unique therapeutic opportunity. This review highlights how phage-antibiotic synergy, particularly when directed at efflux systems, can both restore antibiotic sensitivity and impose evolutionary trade-offs that destabilize resistance mechanisms.

From molecular mechanisms to clinical case reports, emerging evidence supports the feasibility of targeting efflux pumps with tailored phage cocktails, either alone or in combination with antibiotics and efflux pump inhibitors. While challenges remain, including variability in phage-host interactions, resistance emergence, and regulatory complexities, the convergence of mechanistic rationale, clinical proof-of-concept, and translational momentum positions efflux-targeting phage therapy as a viable addition to the antimicrobial arsenal.

Realizing this potential will require continued integration of genomics, real-time susceptibility profiling, and adaptive phage design within personalized treatment frameworks. As the field advances, efflux pumps may shift from being a barrier to therapy to becoming a gateway for innovative, precision-guided infectious disease management.
